# An Uncontrollable, Aggressive Patient at a Free-Standing Emergency Department

**DOI:** 10.7759/cureus.32742

**Published:** 2022-12-20

**Authors:** Joel Crane, Brittney E Aguiar, Jeffrey A Nielson

**Affiliations:** 1 Emergency Medicine, Kettering Health Dayton, Dayton, USA; 2 Emergency Department, Grandview Medical Center, Dayton, USA

**Keywords:** free standing emergency department, frontal lobe injury, temporal lobe injury, aggression, post traumatic brain injury

## Abstract

We present the case of an aggressive male patient who was unable to be successfully sedated with conventional medications in the ED and ultimately required intubation to ensure the safety of the patient himself and the staff. After admission to the ICU, he was found to have atrophy of the frontal and bilateral lobes secondary to a traumatic brain injury (TBI) 19 years prior. Managing the patient required collaboration with the intensivist, hospitalist, and psychiatry and neurology teams for 10 months, and he was refused admission to multiple psychiatric facilities due to safety concerns because of his high level of aggression and unpredictability. An out-of-state, high-security facility eventually accepted the patient. The second challenge was finding a highly trained medical team willing to transport the patient. This case illustrates the difficulty and safety concerns with regard to managing an aggressive patient with previous TBI when the commonly used medications do not produce the desired effect. A literature search did not reveal a standard protocol or consensus on managing these types of patients in emergent situations.

## Introduction

According to the Centers for Disease Control and Prevention (CDC), there are approximately 3.5 million new traumatic brain injury (TBI) cases each year [1]. In the acute care setting, up to 41% of these cases, and up to 71% of patients in rehabilitation units, will display agitation and/or aggression [1]. The aggression and agitation secondary to a TBI have a significant impact on the patient’s quality of life and lifestyle [1]. TBI can lead to problems with interpersonal relationships, employment, and interactions with law enforcement. Patients can also develop delusional disorders and schizophrenic-like psychotic syndromes after a TBI [2]. Delusional disorders usually develop during the first year after the TBI, and schizophrenia-like psychoses develop later at years three to four after the TBI [1]. Patients with a history of substance abuse are at a higher risk for poor outcomes after a TBI [3]. In TBI-related psychotic disorders, MRI and CT show frontal and temporal lobe changes [1,4]. Loxapine is a first-generation antipsychotic that has been found to be effective in the treatment of agitation in an emergency crisis situation [5]. This particular drug is not stocked in smaller EDs and would have to be obtained from the main pharmacy in larger hospitals. Very few medications have been found to be effective in TBI patients in an acutely agitated state [5].

## Case presentation

A 40-year-old male presented to the ED from a senior living facility with aggressive behavior and combativeness. The report from the paramedic stated that the patient had assaulted a nursing assistant and the nursing director, and hence he had been brought to our free-standing ED for evaluation. A chart review revealed that he had recently been evaluated at a nearby ED for aggression. At the outside ED, he had been given haloperidol 5 mg intramuscular (IM) and diphenhydramine 25 mg IM and then placed in four-point restraints. After a few hours of observation, he had been more cooperative and found to be stable to be discharged back to the senior living facility. His medications at the facility had included divalproex 500 mg delayed release daily, chlorpromazine 600 mg every night at bedtime (QHS), memantine 5 mg every 12 hours (Q12), quetiapine 50 mg four times a day (QID), lorazepam 2 mg QID, benztropine 2 mg IM daily, clonidine HCL 0.1 mg twice a day (BID), gabapentin 300 mg QID, temazepam 30 mg QHS, and hydroxyzine pamoate 50 mg three times a day (TID) as needed (PRN). His past medical history included seizure disorder, dementia, insomnia, cortical blindness, personality disorder, epilepsy, behavioral disturbances, genital herpes, TBI after jumping from a moving vehicle 19 years ago, and emotional lability. He also had a history of assaulting staff at multiple facilities. The patient had already been seen by psychiatry and neurology while at the extended care facility (ECF). His surgical history included multiple cranial surgeries, one of which was a bilateral frontal decompressive hemicraniectomy.

Due to continued aggression at the nursing facility, he was then brought to this ED for additional evaluation and treatment. He was initially cooperative for blood tests and a urinalysis. His electrocardiogram (EKG) showed a nonspecific intraventricular conduction delay. His acetaminophen level and salicylate level were undetectable. A drugs of abuse urine test was negative. His thyroid-stimulating hormone (TSH) level was 2.883 ulU/ml, and the ethanol test returned negative. The partial pressure of the carbon dioxide was 39 mmHg.

On the initial presentation, the patient was alert and oriented to person, place, and time. He had no focal weakness and no ataxia. He denied any suicidal or homicidal ideation. His heartbeat was regular, without murmurs, gallops, or ectopy. His lungs were clear bilaterally without wheezing, rhonchi, and rales. The abdomen was soft, nondistended, and nontender throughout. Pulses were equal and strong bilaterally throughout. The patient then became more verbally aggressive while he was awaiting assessment by the behavioral health team. He became impatient and attempted to leave the ED room and started yelling at the staff, and hence he was given haloperidol 5 mg IM and lorazepam 2 mg IM. After about 20 minutes, he was reevaluated and he was again becoming aggressive; he was then given ziprasidone 10 mg IM. After another 20 minutes, his aggression continued to escalate, and he was given 250 mg of ketamine IM. While he was observed for another 30 minutes, staff had to determine how to keep the patient and the staff safe in that small outlying ED with a 20-minute transport time to our closest network hospital. He started to slam his head into the plexiglass window of the psychiatric room, and four community police officers had to be brought in to restrain him.

The decision was made to intubate the patient with etomidate and rocuronium to control the aggressive behavior and for the safety of the patient and hospital staff. The four officers held his arms and legs while rocuronium was given IM. When sedation began to set in, the nurses quickly placed an intravenous (IV) catheter, and etomidate was given. Intubation was done for airway protection and was uneventful. He was then sedated using propofol but was still combative despite using our maximum hospital dose of 50 mcg/kg/min, and vecuronium (dosing regimen) was required for transporting the patient to the ICU at the main hospital via medical flight. Maximum sedation had to be achieved for the flight since any aggressive behavior could put the helicopter and flight crew in danger. After intubation, a chest X-ray was done, which did not show any evidence of infection. A urinalysis was then obtained and found to be normal. The patient's vital signs were as follows - heart rate: 78 beats per minute, respiratory rate: 16 breaths per minute, pulse oximetry: 97% oxygen saturation, and blood pressure: 119/68 mmHg. Since the patient had a history of genital herpes, he was suspected to have herpes encephalitis; however, adequate sedation could not be achieved for a lumbar puncture to obtain a sample of cerebrospinal fluid (CSF). The patient would need to be put under general anesthesia for a lumbar puncture, which was not available at the outlying ED. Even with the propofol infusion and vecuronium boluses, the sedation level was still not adequate to perform a lumbar puncture. Since vecuronium is rarely used in the outlying ED, the available quantity was insufficient for adequate sedation to dose this patient using weight-based dosing.

**Figure 1:**
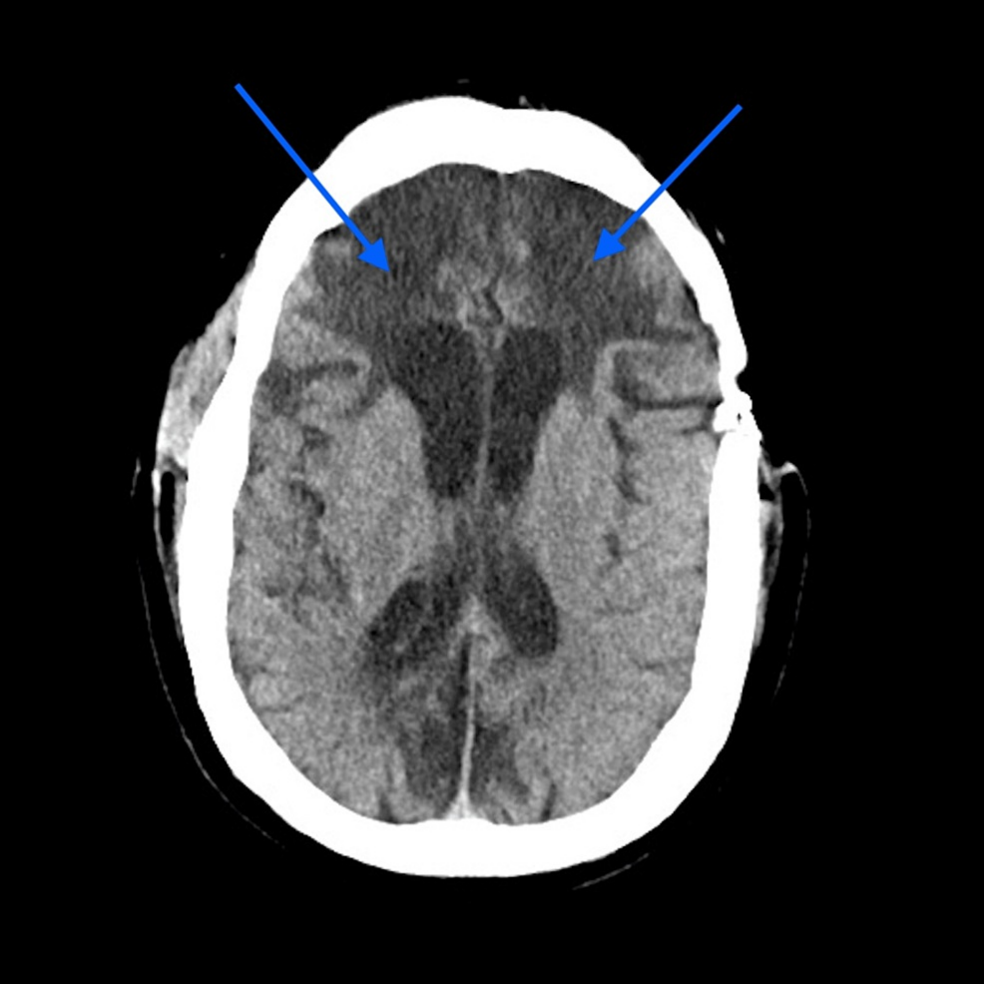
CT of the head - horizontal cut showing frontal lobe atrophy (blue arrows) CT: computed tomography

**Figure 2:**
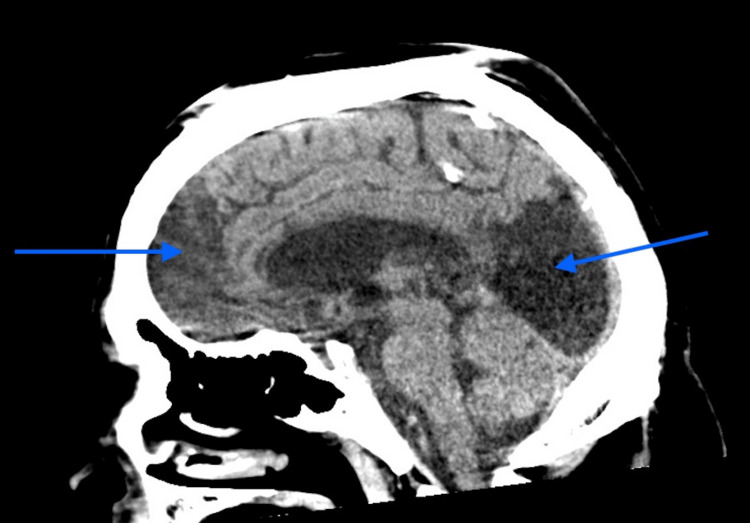
CT of the head - sagittal cut showing frontal and occipital lobe atrophy (blue arrows) CT: computed tomography

At the receiving hospital, a CT of the head was performed, which showed severe atrophy of the frontal lobes bilaterally (Figures 1, 2). After he was extubated in the ICU, the patient was evaluated by psychiatry and neurology. He had already been declined admission to psychiatric units and multiple psychiatric facilities due to safety concerns because of his aggressive behavior. The neurology team got involved and collaborated with the psychiatric team to manage his aggression. He was formally diagnosed with vascular dementia with behavioral disturbance in the setting of chronic TBI. Warfarin was added to his medications since he developed deep vein thrombosis (DVT) during his prolonged hospitalization. He also contracted coronavirus disease 2019 (COVID-19) during the pandemic while in the hospital. His aggression and unpredictability were such a concern that he had a security officer assigned to him throughout his hospital stay. He was ultimately discharged on sodium valproate, levetiracetam, topiramate, olanzapine, perphenazine, propofol, warfarin, haloperidol, clonidine, and memantine. He was deemed too dangerous to be cared for in any local ECFs due to the severity and frequent episodes of aggression. He remained in the hospital for 10 months until he was accepted at an out-of-state, high-security facility.

## Discussion

Agitated patients presenting to the EDs is an increasing problem across the United States [6]. It is important to stabilize the patient primarily, before initiating the investigation into the underlying causes of encephalopathy. The major concern about this case was the level of aggression shown by the patient, which could not be managed by the medications given in the ED. Of note, 50% of medical providers suffer physical violence at some point in their careers [7,8]. A survey showed that two-thirds of physicians have minimal or no training in the management of an agitated patient [7]. Other studies have shown that 20-40% of hospitals do not have training programs for managing combative patients [7]. This case in particular demonstrates the importance of having a broad differential diagnosis for altered mental status while balancing it against the immediate needs of the patient and ED staff; 5-10% of ED visits pertain to altered mental status [7]. In such cases, it is often difficult to obtain a proper history from the patient [9].

Haloperidol and lorazepam were initially used for our patient since these medications are commonly used in the ED for agitation and the staff is familiar with them [7,10]. A second-generation antipsychotic (ziprasidone) was then added to the first-generation antipsychotic (haloperidol) and benzodiazepine (lorazepam) for an additive effect. Ketamine was then chosen for its sedative and dissociative characteristics [9]. When these medications failed to sedate the patient, a decision was made to intubate him for the safety of the patient himself and the staff. It was felt that giving any more medication would have little benefit as the patient kept becoming more aggressive and sufficient time had passed for the medication to take effect.

The inpatient team placed the patient on a variety of medications with varying mechanisms of action to treat his aggression. Sodium valproate increases the effects of gamma-aminobutyric acid (GABA) to serve as an antiepileptic and mood stabilizer [5]. The patient's key issues were his large fluctuations in mood, impulsivity, and seizure disorder. A wide variety of medicines were used in combination to manage these. Levetiracetam, an antiepileptic that selectively prevents hypersynchronization of epileptiform burst firing, was also added for the seizure disorder, but it typically does not affect aggressiveness [5]. Another antiepileptic, topiramate, was added; it blocks voltage-dependent sodium channels, augments GABA activity, antagonizes glutamate receptors, and inhibits carbonic anhydrase. Olanzapine is a second-generation antipsychotic, and it was used to antagonize dopamine and serotonin 5-hydroxytryptamine 2 (HT2) receptors. Perphenazine and Haldol are two other first-generation antipsychotics that were added to antagonize dopamine D2 receptors. Propofol being an anesthetic was used during episodes of severe agitation. Clonidine stimulates alpha-2 adrenergic receptors and is used for attention deficit hyperactivity disorder. This was added to help with his ability to focus and rest. Finally, memantine, which binds N-methyl-D-aspartate receptors and may slow nerve damage, was also used.

Managing acutely psychotic patients, whether due to substance abuse or psychiatric history, constitutes an important part of the duties of an emergency medicine physician. It is critical to be able to administer medications to chemically restrain combative patients. Oftentimes, physical restraints must be used for patient and staff safety. A free-standing emergency department has limited resources to manage these patients. There may not be enough ancillary staff or even medications available to deal with aggressive patients. As free-standing emergency departments become more common, it would be prudent to develop protocols for managing acutely psychotic patients and expediting their transport from outlying facilities to hospitals where consultations with specialists can be arranged.

## Conclusions

Pharmacologically uncontrollable aggression in acutely psychotic adult patients is exceedingly rare. There is a multitude of pharmacologic agents used for these circumstances, and many of them are used in combination. Our patient’s severe aggression still persisted enough for him to require deep sedation and permanent residence at a high-security facility, despite months of medical interventions. With the surge in free-standing emergency departments, which often have limited resources, it is important to have protocols in place to keep patients and staff safe during patient care.
